# Levels of Burnout and Its Determinant Factors Among Nurses in Private Hospitals of Addis Ababa, Ethiopia, Ethiopia, 2020. A Multi Central Institutional Based Cross Sectional Study

**DOI:** 10.3389/fpubh.2022.766461

**Published:** 2022-04-25

**Authors:** Dejen Getaneh Feleke, Ermiase Sisay Chanie, Misganaw Girma Hagos, Behailu Tariku Derseh, Sheganew Fetene Tassew

**Affiliations:** ^1^Department of Pediatrics and Child Health Nursing, College of Health Sciences, Debre Tabor University, Debre Tabor, Ethiopia; ^2^Tigur Ambessa Hospital, Addis Ababa University, Addis Ababa, Ethiopia; ^3^Department of Public Health, College of Health Sciences, Debre Birhan University, Debre Birhan, Ethiopia; ^4^Department of Emergency Medicine and Critical Care Nursing, College of Health Sciences, Debre Tabor University, Debre Tabor, Ethiopia

**Keywords:** burnout syndrome, nurses, Maslach burnout inventory, Ethiopia

## Abstract

**Background:**

Burnout among nurses is a significant problem in healthcare establishments and has negative implications on clinical outcomes. International studies have shown the prevalence of burnout ranged from 10 to 70%. However, this is unknown among nurses in private hospitals in Addis Ababa. The study was intended to assess the levels of burnout and the associated factors among nurses working in private hospitals in Addis Ababa, Ethiopia, 2020.

**Methods:**

An institution-based cross-sectional study was used. A probability sampling, specifically, a simple random sampling technique was employed to collect data, and the Maslach burnout inventory human services survey (MBI-HSS) instrument was adapted to measure the levels of burnout. The data obtained was edited manually and entered into EPI-data version 4.6 and then exported to SPSS version 25 for analysis. Logistic regression was used to identify the association between the dependent and independent variables and variables with *p* < 0.25 on bivariate analysis were taken into multivariate logistic regression, and then variables with *p* < 0.05 were considered statistically significant.

**Result:**

A total of 385 questionnaires were distributed to participants, but only 368 (96%) of them were collected and included in this study. The majority 56% and 69.8% of them were females and belonged to the age group of 20–29 years, respectively. Two hundred seven (56.5%) of them reported suffering from a high level of burnout. In the multivariate logistic regression analysis, night duty shift [AOR = 2.699; 95% CI: (1.043–6.987)], excessive workload [AOR = 6.013; 95% CI: (3.016–11.989)], staff shortage [AOR = 6.198; 95% CI: (3.162–12.147)], persistent interpersonal conflict [AOR = 2.465; 95% CI: (1.225–4.961)], and nurses' poor health status [AOR = 3.4878; 95% CI: (1.815–8.282)] demonstrated a statistical significant association with the professional burnout.

**Conclusions and Recommendations:**

Nurses' burnout in private hospitals of Addis Ababa was highly prevalent. Therefore, ensuring adequate staffing and minimizing the workload of nurse professionals are mandatory to prevent it.

## Introduction

Burnout is conceptualized as a psychological syndrome that occurs in response to interpersonal stressors in the work environment. It is an individual-level phenomenon and can be viewed as a negative emotional experience which is a chronic, ongoing affective response (1). Burnout syndrome does not have immediate manifestations, but appears as a gradual reaction of emotional breakdown due to the prolonged exposure to stress factors, which leads to an increase in dehumanization levels and professional dissatisfaction (2).

Nursing burnout is more likely related to job demands, and burned out health professionals believed that their objectives are not achieved, which is accompanied by physical depletion, feelings of helplessness, disillusionment, decreased motivation, dysfunctional behavior, negative self-concepts, and negative attitudes toward work and life itself. To prevent further energy depletion, employees distance themselves mentally from their work by developing depersonalizing attitudes. Due to this reason, their work performance tends to diminish and they may feel incompetent and in efficacious, leading to a decrease in the quality of the health care facilities, which ultimately affects the satisfaction of the patient. Most of the time, burnout can occur due to a widening gap between the individual and demands of the job (3).

Burnout can affect every occupation. However, it is thought to be more prevalent among human professionals as the services offered by them are in response to the needs of the society. Among health professionals, professional nursing, in particular, is a service for the promotion of human and social welfare. Comparatively, nurses are the first line of contact; they spend the most time with patients and are constantly exposed to the emotional strains of dealing with the sick and dying. Such stressors when left unchecked lead to burnout. Burnout syndrome among nurses in hospitals has become a worldwide phenomenon that negatively impacts the quality of care, safety of patients and working staff. An estimated range of 10–70% of nurses are affected by burnout throughout the world (4, 5).

In a multi-country, cross-sectional study conducted in 10 European countries among staff nurses, high levels of burnout were reported in England (42%) and low levels of burnout were in Switzerland (15%) (6). Moreover, nurses' burnout is a real concern in African hospitals. A high prevalence rate of burnout, around 70%, was reported in Senegal and Malawi. In Tunisia, two nurses out of three are affected by burnout, while in Morocco it affects one in every two nurses working in hospitals (7). Nurses' burnout has led to emotional exhaustion as well as a loss of compassion for others (depersonalization) and a sense of low personal accomplishment due to the presence of job demands like heavy work overload; lack of social support from management, supervisor, or colleagues; limited promotion; nurse–patient ratio imbalance; confronting with the death of patients; shift work; and being underpaid (1).

Burnout has been associated with reduced organizational efficiency and work-related problems such as low morale, poor quality of care, lowered productivity, absenteeism, interpersonal problems, and employee turnover (6). A 10% increase in nurse's turnover results in a 9.4–17.4% increase in the discharge death rate due to nurse burnout (8). The above situation may lead to such an extent that can affect the reputation of the health care facilities because, in most cases, health care facilities are evaluated by communities through the quality of health care they provide and the trust that can be placed into its professionals.

Despite the existence of numerous studies conducted in Ethiopia on the prevalence and associated factors of burnout among nurses who are working in public hospitals, as far as our knowledge, there is found a lack of studies conducted in private hospitals in Addis Ababa administration. Although the healthcare industry in Addis Ababa expanded drastically, this expansion has taken place mostly in the private sector. Many hospitals are established throughout the city which opened employment opportunities for the professionals in large numbers, but at the same time could not meet the demand of the number of hospitals required to provide better services to the people at large. In private hospitals, a high number of patients is seen in every hospital from morning till late evenings and this increases the number of duties of the nursing staff because most of the private hospitals in Addis Ababa cover many tasks with the minimum number of nurses. The current duty shift during data collection period was alternate, day, night. In our study, the nurses perception about the presence of work-load burnout was 79.7%. The variation of burnout across the various nursing disciplines/departments were the nurses' perception about the presence of work load, nurses' perception about the presence of nursing staff shortage, the presence of persistent interpersonal conflicts, nurses' perception about the management of their organization's support nursing staff, whether or not they got any professional recognition, respect, or reward from their hospital administrator. The information from the study result provided input for the planner and policymaker to consider the extent of the problem and develop a well-standardized guideline and policy to tackle the problems that cause burnout among nurses to improve its work-related consequences. To address the gap in the research for various health care institutions, the study was intended to assess burnout syndrome and its determinant factors among nurses in private hospitals of Addis Ababa, Ethiopia.

## Methods and Materials

### Study Area and Period

The study was conducted at private hospitals in AA city. Addis Ababa is the capital city of Ethiopia and seat of the Africa Union. It is the largest city in Ethiopia, with a population of 3,475,952 according to the 2007 population census with an annual growth rate of 2.7%. In Addis Ababa, there are around 40 private hospitals that provide health care services not only for Addis Ababa residents but also serve as referral facilities for the nation. Out of these ten hospitals (Korea Hospital, Nordic Hospital, T/Haymanot Hospital, Haleluya Hospital, Bethezatha Hospital, Kadisco Hospital, Yerer Hospital, Addis Hiwot Hospital, Landmark Hospital, and Girum Hospital) were selected for this study, and data collection working units were ward (inpatient department), intensive care unit, emergency department, outpatient department, and operation Room. The study was conducted from 1 November to 30 December 2019.

### Study Design and Participant's Characteristics

An institutional based cross-sectional study was employed. For this study, the source population was all nurses who are working in Addis Ababa private hospitals. The study population was all nurses who are working in different departments in selected private hospitals of Addis Ababa, which fulfill the inclusion criteria. All nurses who are assigned in the departments with work experience of 12 months and above were included in this study.

### Sample Size Determination and Sampling Procedure

To determine the sample size, the following assumption was used. Prevalence and determinant factors of nurse's burnout were taken from a previous related study which is 54.8%, with confidence levels 95%, and 5% margin of error (9).


$$\begin{array}{l} n=\frac{{\left(1.96\right)}^{2*}0.548\;\left(1-0.548\right)}{{\left(0.05\right)}^{2}} \end{array}$$

where *n* = 380.6 ≈ 381. Since the total population was 4,273 nurses, which is <10,000, we used the correction formula: nf = $\frac{ni}{1+\frac{ni}{N}}$, where; nf = final sample size, ni = initial sample size, *N* = total population (10, 11). Applying the values, nf = $\frac{381}{1+\frac{381}{4273}}$, *n* = 349.8 ≈ 350.

The non-response rate (absenteeism and refusal) was taken to be 10% using previous related research response rate = 350^*^0.1 = 35, then (350 + 35), the final sample size was 385. To achieve the representativeness of the study participant simple random sampling technique was employed to draw the sample size from 40 private hospitals currently functioning in Addis Ababa. Due to the scarcity of resources, only ten hospitals were selected by the lottery method and considered to participate in the study. The ten hospitals were (Korea hospital 248 nurses, Nordic hospital 101 nurses, T/Haymanot hospital 88 nurses, Halelujah hospital 104 nurses, Bethzatha hospital 51 nurses, Kadisco hospital 86 nurses, Yerer hospital 65 nurses, Adishiwot hospital 43 nurses, Landmark hospital 47 nurses, and Girum hospital 91 nurses). Then the final sample was selected from the respective hospitals by using proportional to size allocation formula:


$$\begin{array}{l} \frac{ni^{*}nf}{N} \end{array}$$

where; ni: number of nurses in each selected hospital, nf: final sample of the study, *N*: total number of nurses in the selected hospitals.

Korea hospital = 248^*^385/924 = 103, Nordic hospital = 101^*^385/924 = 42, T/Haymanot hospital = 88^*^385/924 = 37, Halelujah hospital = 104^*^385/924 = 43, Bethzatha hospital = 51^*^385/924 = 21, Kadisco hospital = 86^*^385/924 = 36, Yerer hospital = 65^*^385/924 = 27, Addis Hiwot hospital = 43^*^385/924 = 18, Landmark hospital = 47^*^385/924 = 20, Girum hospital = 91^*^385/924 = 38.

Then systematic sampling was performed to select samples from each hospital by using the formula K = *N*/*n*, where *N*: the population sample size in the selected hospital, n: required sample size, and K: interval.

Korea hospital = 248/103 = 2.4 ≈ 2, Nordic hospital = 101/42 = 2.4 ≈ 2, T/Haymanot hospital = 88/37 = 2.37 ≈ 2, Halelujah hospital = 104/43 = 2.42 ≈ 2, Bethzatha hospital = 51/21 = 2.43 ≈ 2, Kadisco hospital = 86/36 = 2.39 ≈ 2, Yerer hospital = 65/27 = 2.41 ≈ 2, Adishiwot hospital = 43/18 ≈ 2, Landmark hospital = 47/20 = 2.35 ≈ 2, and Girum hospital = 91/38 = 2.39 ≈ 2. Therefore, the selection was every second unit in the population in each hospital from the nurse's roster.

### Data Collection Method and Procedure

Data were collected through 24 h, both in working time and night duty time. Data collection was conducted by the trained data collectors and the supervisor. Training was given to data collectors and supervisors for 1 day. Two B.Sc. data collectors and one M.Sc. supervisor were recruited based on previous experience of data collection. After identifying the study subjects, informed consent was obtained to confirm willingness, and confidentiality was ensured to all the study subjects and the self-administered structured questionnaire was administered. Non-respondents were encouraged to fill in the questionnaire and were revisited at least twice. The respondents were encouraged to respond to all items in the questionnaire within the time they devoted as much as possible to minimize a large non-response rate.

### Data Collection Tool

A self-administered standardized questionnaire was adapted by the principal investigator after reviewing different related literature. The questionnaire has two sections that is used to obtain information relevant to the study: the first section asked about participant's personal information which includes sociodemographic, work related, and organizational related factors of an individual. The second section was a standard question used to assess the levels of burnout, the English version of Maslach's Burnout Inventory Human Services Survey (MBI-HSS), which comprises 22 items regrouped into 3 sub-scales: emotional exhaustion (EE; nine items), depersonalization (DP; five items), and personal accomplishment (PA; eight items). Each item was answered on a 7-point Likert scale ranging from “never” (=0) to “daily” (=6). The results of the inventory consisted of three separate scores, one for each factor or subscale. A combination of high scores on EE and DP, and a low score on PA, were considered to correspond to a high level of burnout. Scores were considered high if they are in the upper third of the normative distribution, middle if they are in the middle third, and low if they are in the lower third. The MBI-HSS was a self-administered questionnaire, which has been reliable (reported 0.83 with a Cronbach's alpha method) in the pretest.

### Study Variables

#### Dependent Variable

Burnout syndrome.

##### Independent Variables

**Sociodemographic characteristics:** age, sex, marital status, educational qualification, years of work experience, salary/wages.

**Work-related factors:** work load, working unit, inter-personnel conflict, and night shift duty.

**Organizational factors:** lack of management support, lack of professional recognition, respect, or reward, and personal/material resources.

### Measurements of Variables

Emotional exhaustion: low (≤16), moderate (17–26), high (≥27).

Depersonalization: low (≤6), moderate (7–12), high (≥13).

Personal accomplishment: low (≤31) moderate (32–38), high (≥39) (9).

### Operational Definition

**Burnout:** It is a psychological condition characterized by emotional exhaustion (EE), depersonalization (DP), and low personal achievements (LPA) (12).

**Emotional exhaustion:** Leading indicator of burnout and defined as feelings of fatigue and of being drained by one's work.

**Depersonalization:** The negative attitude toward and a dehumanizing treatment of one's clients in the workplace (having a negative view about their clients).

**Low personal accomplishment:** Reduced competence and achievements in one's work a feeling of lower capability in doing personal duties) (9).

**High levels of burnout:** High scores on emotional exhaustion (EE) and depersonalization (DP) subscales, and a low score on personal achievement (PA) subscale.

**Moderate level of burnout**: Represents burnout score an average number on three dimensions [emotional exhaustion (EE), depersonalization (DP) subscales, and personal achievement (PA) of subscale].

**Low levels of burnout**: Low scores on emotional exhaustion (EE) and depersonalization (DP) subscales, and a high score on personal achievement (PA) subscale (13).

### Data Processing and Analysis

The data was checked for its completeness, cleaned, entered, and coded into EPI-data version 4.6 computer programs to minimize data entry errors. The data that are appropriate for analysis were exported to SPSS version 25 and the analysis was done. The outcome (dependent) variable was measured on a dichotomous scale and had multiple independent variables; it used logistic regression. Prior to performing regression analysis, data were checked for multicollinearity, normality, linearity, independence of residuals and outliers. No major violations of the assumptions of the regression analysis were found. Model adequacy was checked by omnibus tests of coefficients and Hosmer-Lemeshow goodness-of-fit test. The squared multiple correlation coefficients were 0.564, which was between zero (0) and one (1), and indicated that 56.4% of the variation in an outcome variable is explained by the variation in the predictor variables. To explain the study population in relation to relevant variables, descriptive statistics such as frequencies and percentages were calculated. The data were analyzed by using logistic regression with step-wise model selection method to identify the association between burnout and determinant factors. To control confounder, all predictors that have been associated with the outcome variable on bivariate logistic regression with a *p* < 0.25 were included in the logistic regression model of multivariate analysis and presented in the table. For all analysis a *p* < 0.05 is considered statistically significant in all the cases.

### Data Quality Assurance

To maintain the reliability of the questionnaire, Cronbach's alpha test was done and reliability reported at 0.83, 0.76, and 0.80 for emotional exhaustion, depersonalization, and personal accomplishment sub-scale, respectively. The quality of data was assured through careful design and pretesting of the questionnaire. A pretest was conducted on 5% of the sample size on the same source population in Yordanos hospital. The result of the pretest was analyzed and modification was made prior to the actual data collection. A 1-day training was given for both the data collectors and supervisor on the objective of the study and methods of data collection. The supervisor and principal investigator closely followed the day-to-day data collection process and ensured completeness and consistency of the collected questionnaires on a daily basis.

### Ethical Consideration

Ethical clearance was obtained from the Ethical Review Committee of Debre Berhan University, Institution of Medicine and Health Sciences, College of Health Sciences prior to beginning of the study. A letter of cooperation to secure permission of access was given to the selected hospitals included in the study. After obtaining permission from the hospital directors and unit coordinators, informed (written) consent was obtained from the study subjects who have equal chance to participate in the study. To ensure the autonomy of the study participants, their willingness prior to their participation was confirmed after explaining the objective of the study. Information obtained from individual participants was kept secure and confidential. Names and other identifying data of respondents were made anonymous or eliminated throughout the study process to maintain confidentiality.

## Results

### Socio-Demographic Characteristics of the Respondents

A total of 385 questionnaires were distributed to the nurses working in ten different private hospitals in different departments, three hundred sixty-eight (96%) participants responded to the questionnaires and were considered for the study. Two hundred six (56.0%) of the respondents were females. Majority (69.8%) of them belonged to the age group of 20–29 years. With respect to the respondent's marital status, 205 (55.7%) were single. With regard to their educational status, 291 (79.1%) were B.Sc. in nursing. The result illustrates that in regard to years of work experience of the respondents in their facilities, 152 (41.3%) had between 3 and 5 years of work experience. With regard to the working unit, 179 (48.6%) were in inpatient departments (ward). In the duty shift, 236 (64.1%) were in alternate shifts, whereas, the rest 56 (15.2%) were in the night shift. Participants were asked a question, “Do you perceive satisfied with the current monthly salary?” Three hundred forty-six (94.0%) of the respondents were dissatisfied with their current monthly salary. Participants were asked a question, “Do you think to leave the current working unit within the next 12 months related to your work?” Two hundred nine (56.8%) of them reported having the intention to leave their current working unit. Participants were asked a question, “How do you perceive your current health status?” One hundred and fifty-eight (42.9%) of the respondents perceived their health status as fair and 135 (36.7%) as good, while the rest 75 (20.4%) perceived their health status as poor. Participants were asked a question, “How do you perceive health problems have you experienced in relation to your work?” One hundred and seventy-nine (48.6%) of the respondents perceived as experiencing backache, 110 (29.9%) had depression, and 108 (29.3%) had headache. While the rest, 28 (7.6%) and 2 (0.5%) were perceived as experiencing insomnia and hypertension, respectively (see Table 1).

**Table 1:** Socio-demographic characteristics of respondents in private hospitals of Addis Ababa, 2020.

**Variables**	**Category**	**Frequency**	**Valid percent**
Sex	Female	206	56.0
	Male	162	44.0
Age	20–29	257	69.8
	30–39	89	24.2
	40–49	22	6.0
Marital status	Single	205	55.7
	Married	157	42.7
	Divorced	6	1.6
Educational level	B.S.c	293	79.1
	Diploma	63	17.1
	M.S.c	14	3.8
Work experience	3–5 years	152	41.3
	≤ 2 years	126	34.2
	6–10 years	66	18.0
	11–15 years	24	6.5
Working unit	Ward	179	48.6
	ICU	82	22.3
	ED	64	17.4
	OPD	24	6.5
	OR	19	5.2
Current duty shift	Alternate	236	64.1
	Day	76	20.7
	Night	56	15.2
Nurses perception about their salary	Dissatisfied	346	94.0
	Satisfied	22	6.0
Intention to leave current unit within the next 12 months related to your work	Yes	209	56.8
	No	159	43.2
Nurses perception about their current health status	Fair	158	42.9
	Good	135	36.7
	Poor	75	20.4
Nurses perception on health problems they experienced	Bache ache	179	48.6
	Headache	108	29.3
	Depression	110	29.9
	Insomnia	28	7.6
	hypertension	2	0.5

### Nurses Levels of Burnout

Two hundred seven (56.5%) of the participants were suffering from high levels of burnout (PB) during the study period. Two hundred nine (56.8%), 84 (22.8%), and 75 (20.4%) of them had high, moderate, and a low scale of emotional exhaustion (EE), respectively. On the other hand, 207 (56.3%), 94 (25.5%), and 67 (18.2%) of them had high, moderate, and low scale of depersonalization (DP), respectively. Regarding the scale of personal accomplishment (PA), 79 (21.5%), 82 (22.3%), and 207 (56.3%) of them rated as having high, moderate, and low scores, respectively.

### Determinants of Nurse's Burnout

To investigate the association of independent variables with burnout, both bivariate and multivariate analyses were used. Those variables that showed association with outcome variables in the bivariate analysis (at *p* < 0.25) were selected as candidate variables for multivariable logistic regression analysis. Nurses age, educational level, service area, service year, current duty shift, presence of work overload, presence of interpersonal conflict, staff nurses shortage, presence of management support, professional recognition/respect or reward, intention to leave within 12 months, nurses perception about their current health status, and depression were significantly associated with nurse professional burn out (at *p* < 0.25). All variables that have association (at a significance level of 0.25) with the outcome variables in bivariate logistic regression analyses were included in the multiple logistic regression models. After controlling for the effects of potentially confounding variables using multiple logistic regression, duty shift, workload, staff nurse's shortage, interpersonal conflict, and nurse's perception about their current health status were significantly associated with nurse professional burnout (at *p* < 0.05).

Nurses who were working in night duty shifts were 2.7 times [AOR = 2.699; 95% CI: (1.043–6.987)] more likely to develop professional burnout as compared to those who were working at day duty shift, and nurses who were working at alternate duty shifts were 81% [AOR = 0.193; 95% CI: (0.065–0.570)] less likely to develop professional burnout as compared to those who were working in day duty shifts. Nurses who had suffered from excessive work overload [AOR = 6.013; 95% CI: (3.016–11.989)] and not working with the standard proportion [AOR = 6.198; 95% CI: (3.162–12.147)] were comparatively 6 times more likely to develop professional burnout. Moreover, burnout levels were comparatively 2 times [AOR = 2.465; 95% CI: (1.225–4.961)] higher among nurses those who had persistent interpersonal conflict at their workplace. In addition to the above factors, staff nurses those who had perceived their health status as poor [AOR = 3.4878; 95% CI: (1.815–8.282)] and fair [AOR = 2.863; 95% CI: (1.171–7.003)] were 3.8 and 2.8 times more likely to develop professional burnout as compared to staff nurses who had perceived their health status as good, respectively (see Table 2).

**Table 2:** Bivariate and multivariate analysis of factors associated with nurses' levels of burnout in Addis Ababa, 2020.

**Variables**	**Nurse burnout**		**COR (95% CI)**	**AOR (95% CI)**	***P*-value**
	**Yes**	**No**			
**Age**					
20–29	149	108	2.959 (1.166–7.519)	2.370 (0.217–25.932)	0.480
30–39	51	38	2.873 (1.068–7.752)	4.884 (0.524–45.530)	0.164
40–49	7	15	1	1	
**Educational level**					
BSc	171	120	1.783 (1.029–3.086)	3.616 (0.526–24.853)	0.191
Diploma	28	35	1	1	
**Service area**					
Ward	75	104	1	1	
ED	47	17	3.834 (2.045–7.194)	1.271 (0.197–8.197)	0.801
ICU	68	14	6.736 (3.521–12.821)	3.831 (0.770–19.231)	0.101
**Service year**					
< /=2 years	76	50	3.040 (1.211–7.631)	3.384 (0.313–36.567)	0.315
3–5	88	64	2.747 (1.110–6.803)	2.513 (0.234–27.010)	0.447
6–10	35	31	2.257 (0.850–5.988)	1.744 (0.194–15.662)	0.620
11–15	8	16	1	1	
**Duty shift**					
Day	19	57	1	1	
Night	45	11	12.345 (5.291–28.571)	2.699 (1.043–6.987)	0.041*
Alternate	143	93	4.608 (2.577–8.264)	0.193 (0.065–0.570)	0.003*
**Nurses perception about presence of work load**					
Yes	165	50	8.696 (5.435–14.085)	6.013 (3.016–11.989)	0.000*
No	42	111	1	1	
**Nurses perception about presence of nursing staff shortage**					
Yes	171	36	12.631 (7.666–20.810)	6.198 (3.162–12.147)	0.000*
No	44	117	1	1	
**Presence of persistent interpersonal conflict**					
Yes	115	58	2.222 (1.456–3.390)	2.465 (1.225–4.961)	0.011*
No	92	103	1	1	
**Nurses perception about mgt**.					
**of their organization support nursing staff**					
Yes	26	32	1	1	
No	181	129	1.727 (0.982–3.037)	2.715 (0.707–10.429)	0.146
**Nurses perception on whether**					
**or not got any prof. recognition, respect**					
**or reward from their hospital administrator**					
Yes	13	17	1	1	
No	194	144	1.761 (0.829–3.745)	0.975 (0.159–5.969	0.978
**Nurses perception about their current health status**					
Poor	56	19	4.292 (2.299–8.000)	3.878 (1.815–8.282)	0.000*
Fair	96	62	2.252 (1.408–3.600)	2.863 (1.171–7.003)	0.021*
Good	55	80	1	1	
**Leave current unit within**					
**the next 12 months related to your work**					
Yes	124	85	1.335 (0.881–2.024)	0.998 (0.487–2.046)	0.996
No	83	76	1	1	
**Nurses perception on**					
**whether experience depression**					
Yes	73	37	1.825 (1.147–2.907)	1.426 (0.689–2.950)	0.339
No	134	124	1	1	

NB: p-value <0.05^*^, AOR, adjusted odd ratio; COR, crude odd ratio.

## Discussion

To successfully tackle burnout problems in Ethiopia, particularly in the present study area, there appears a need to investigate the level of burnout and associated factors among nurses. The finding of this study showed that from the total, 56.5% of nurses who were working in Addis Ababa private hospitals experienced burnout. Among the study participants, 56.8 and 56.3% were high on emotional exhaustion (EE) and depersonalization (DP) sub-scale, respectively. On the other hand, 56.3% was low on the personal accomplishment (PA) sub-scale.

Nurses' level of burnout in this study was higher compared to a previous finding reported in Thailand and Singapore, which were 50.2 and 55%, respectively (14, 15). Furthermore, the level of burnout in this study was higher than the findings reported from the two multi-national studies conducted in Saudi Arabia, which revealed 45 and 45.9% (16). Moreover, this study finding is lower than the study conducted in Lebanese nurses in regard to emotional exhaustion (EE) but higher in regard to depersonalization (DP) and personal accomplishment (PA) sub-scale which were 77.5, 36, and 33%, respectively (17). Additionally, this finding was higher compared to a report from South Africa which was 16, 13, and 10% high on emotional exhaustion (EE) and depersonalization (DP) sub-scale and low on personal accomplishment (PA) sub-scale, respectively (18). The possible reason for the difference might be due to an imbalance between nurses and patient ratio which increases the responsibility, level of duty, and stress on nurses. The second aim of this study was to determine whether there were determinant factors (sociodemographic, work-related, and organization-related factors) significantly associated with burnout status the participants experienced. None of the sociodemographic variables used under the study, such as age, sex, marital status, work experience, educational qualifications, and working area, showed any significant difference in terms of their burnout score. These findings were in line with a previous study conducted in Delhi (10). Moreover, these findings were also supported by a previous study done in Palestine with regard to educational level and burnout (18). Nonetheless, these findings are consistent with the previous studies done in Portugal, Brazil, US, and Greece (12, 19–21).

In this study, a work-related factor has an association with the nurse's professional burnout, which was in the selected private hospitals, for example, nurses who had night duty shifts were significantly associated with burnout as compared to day duty shift. This finding is consistent with the findings of previous studies done in Portuguese (3). The possible reason might be working at night shift disturbs an individual's circadian cycle and their rest and sleep. Those working in night shifts have to sleep in the daytime, when it is not possible to have a deep and good quality sleep. It affects the individual's physiologic balance. Excessive work overload was also found to be one of the strong predictors of burnout. Nurses, those who perceive the presence of excessive workload in their respective units, were significantly more vulnerable to develop professional burnout than their counterparts. This finding is consistent with the findings of previous studies done in America, China, Saudi Arabia, and India (17, 22–24). The possible reason might be the absence of a clear job description; nurses who are working at selected private hospitals were prone to extraordinary practice other than the nursing care, i.e., professionally nurses act as doctors, laboratory technicians, pharmacists, physiotherapists, nutritionists, and mechanically they act as a porter, oxygen technician, cleaner, operator, etc. The presence of persistent interpersonal conflict at the work place was significantly associated with nurse professional burnout than their counterparts. This finding is supported by the previous study done in Australia (25).

In this study, the organization-related factor has an association with the nurse's professional burnout, which was the selected private hospitals, where staff nurses' shortage has significant association with professional burnout in the study population. Nurses who had not worked with standard proportion were more vulnerable to experience professional burnout than their counterparts. This finding is consistent with the findings of previous studies done in the USA and South Africa (22, 26). The possible reason might be the majority of selected private hospitals of Addis Ababa cover their many tasks with minimum nursing staff.

In addition to the above factor's burnout is significantly associated with nurse's perception about their health status. Perception of the health status of the participant nurses, indicating that nurses who perceived their health status as poor have high levels of burnout and vice versa. Decreased wellbeing among nursing staff was associated with burnout syndrome. This finding is supported by a study done in Lebanese and South Africa (26, 27). It is obvious that there exists a health issue whenever it can be an indicator of the presence of burnout among professionals.

### Strength

The strength of the present study is that it utilized a strong theoretical basis, using reliable and valid instruments to collect data.

### Limitation

The primary limitation of the study is that this study is a cross-sectional study design and can only reflect experiences of nurses at the time of assessment, and therefore, a causal relationship cannot be established between burnout and its predictors.

The other limitation of the study is the utilization of self-reported measures, which may increase the possibility of response bias.

## Conclusion

This study presents strong evidence that a significant proportion of nurses working in private hospitals in Addis Ababa experienced high levels of burnout. There was noted a significant association identified between nurse's burnout with certain work profile (work load, duty shift, and interpersonal conflict), organizational factor (nursing staff shortage), and nurses' perception about their health status. On this ground, it can be concluded that a lot of reforms are required at the organizational and work environment levels.

### Recommendations

Based on the results of this study, the following recommendations are forwarded by the researcher.

#### For Policymakers

Reforms for the human resources for health: One of the major recommendations is for the policymakers to increase the nursing strength as per the current patient load of the hospital. This requires a time-to-time revising of the nursing positions in hospital as per emerging demands, that is, reforms in terms of recruitment rules.

Clear job description: Clear-cut guidelines for nursing duties and role clarification at all the levels of staff nurse help them prevent vulnerable to extraordinary pressure at the workplace.

#### For Hospitals Administrator

The hospital's nursing managers and authorities in different levels should be paid for making a friendly communication with the staff, team work encouragement, and reducing occupational conflicts at workplace.

They should ensure adequate staffing of nurses to minimize their workload.

They should reduce the number of night duty shifts of staff nurses and make alternate shifts.

Finally, further and rigorous studies about levels of burnout among nurses in private hospitals in Addis Ababa using other study designs are recommended.

## Data Availability Statement

The raw data supporting the conclusions of this article will be made available by the authors, without undue reservation.

## Ethics Statement

Ethical clearance was obtained from Ethical Review Committee of Debre Berhan University, Institution of Medicine and Health Sciences, College of Health Sciences prior to beginning of the study. Written informed consent for participation was not required for this study in accordance with the national legislation and the institutional requirements.

## Author Contributions

DF worked on designing the study, training and supervising the data collectors, interpreting the result, and preparing the manuscript. EC, MH, BD, and ST played their role in analyzing and interpreting the result and wrote the manuscript. All authors were involved in reading and approving the final manuscript.

## Conflict of Interest

The authors declare that the research was conducted in the absence of any commercial or financial relationships that could be construed as a potential conflict of interest.

## Publisher's Note

All claims expressed in this article are solely those of the authors and do not necessarily represent those of their affiliated organizations, or those of the publisher, the editors and the reviewers. Any product that may be evaluated in this article, or claim that may be made by its manufacturer, is not guaranteed or endorsed by the publisher.

## Abbreviations

AA: Addis Ababa
B.Sc: Bachelor of Science
BOS: Burnout Syndrome
CNE: Continuous Nursing Education
DP: Depersonalization
ED: Emergency Department
EE: Emotional Exhaustion
ICU: Intensive Care Unit
MBI-HSS: Maslach's Burnout inventory Human Service Survey
M.Sc.: Master of Science
NGO: Non-Governmental Organizations
SPSS: Statistical Package for Social Sciences
USA: United States of America.
